# A Large Family of *AvrLm6*-like Genes in the Apple and Pear Scab Pathogens, *Venturia inaequalis* and *Venturia pirina*

**DOI:** 10.3389/fpls.2015.00980

**Published:** 2015-11-17

**Authors:** Jason Shiller, Angela P. Van de Wouw, Adam P. Taranto, Joanna K. Bowen, David Dubois, Andrew Robinson, Cecilia H. Deng, Kim M. Plummer

**Affiliations:** ^1^Animal, Plant and Soil Sciences Department, AgriBio, AgriBiosciences Research Centre, La Trobe University, MelbourneVIC, Australia; ^2^School of BioSciences, University of Melbourne, ParkvilleVIC, Australia; ^3^Plant Sciences Division, Research School of Biology, The Australian National University, CanberraACT, Australia; ^4^The New Zealand Institute for Plant and Food Research LimitedAuckland, New Zealand; ^5^Life Sciences Computation Centre, Victorian Life Sciences Computation Initiative, MelbourneVIC, Australia

**Keywords:** effector, Dothideomycete, gene families, RIP, WGS

## Abstract

*Venturia inaequalis* and *V. pirina* are Dothideomycete fungi that cause apple scab and pear scab disease, respectively. Whole genome sequencing of *V. inaequalis* and *V. pirina* isolates has revealed predicted proteins with sequence similarity to AvrLm6, a *Leptosphaeria maculans* effector that triggers a resistance response in *Brassica napus* and *B. juncea* carrying the resistance gene, *Rlm6*. *AvrLm6*-like genes are present as large families (>15 members) in all sequenced strains of *V. inaequalis* and *V. pirina*, while in *L. maculans*, only *AvrLm6* and a single paralog have been identified. The *Venturia AvrLm6*-like genes are located in gene-poor regions of the genomes, and mostly in close proximity to transposable elements, which may explain the expansion of these gene families. An *AvrLm6*-like gene from *V. inaequalis* with the highest sequence identity to *AvrLm6* was unable to trigger a resistance response in *Rlm6*-carrying *B. juncea*. RNA-seq and qRT-PCR gene expression analyses, of in planta- and *in vitro*-grown *V. inaequalis*, has revealed that many of the *AvrLm6*-like genes are expressed during infection. An *AvrLm6* homolog from *V. inaequalis* that is up-regulated during infection was shown (using an eYFP-fusion protein construct) to be localized to the sub-cuticular stroma during biotrophic infection of apple hypocotyls.

## Introduction

Effectors are generally small proteins secreted by plant pathogens that can interact with the host during infection. They may serve to facilitate infection, often by manipulating the host, or may be recognized by host receptor proteins, either directly or indirectly, leading to a resistance response (Chisholm et al., 2006; Jones and Dangl, 2006; Dodds and Rathjen, 2010). The outcome of the plant–pathogen interaction depends on the evolutionary context; the pathogen is under selection pressure to evade detection by the host, while the host must maintain the ability to detect the pathogen. These gene-for-gene relationships are at play in the host specificity of *Venturia inaequalis* races to different *Malus* cultivars, where so far 17 gene-for-gene relationships have been described (Bus et al., 2011). While two resistance (*R*) genes governing race-cultivar specificity have been identified in *Malus* (Belfanti et al., 2004; Schouten et al., 2013) none of the cognate fungal avirulence genes have been isolated. Genomic (BioProject ID PRJNA261633 for *V. inaequalis* and JEMP01000000 for the *V. pirina* genome) and transcriptomic (Thakur et al., 2013) sequences are now available for several isolates of *V. inaequalis* and a *V. pirina* isolate, providing a promising new avenue of bioinformatic discovery to identify effectors; however, identifying specific race-governing effectors of *V. pirina* and *V. inaequalis* among these large predicted secretomes remains a challenge. Most effectors identified to date, in other fungi, are small, secreted proteins (SSPs) with high cysteine content (Stergiopoulos and de Wit, 2009) and *in silico* prediction of effector candidates has been informed by these attributes (Saunders et al., 2012; Guyon et al., 2014; Sperschneider et al., 2015). Effectors are often race- or species-specific (Stergiopoulos and de Wit, 2009); however, it has been reported that some effectors can be more widely conserved, even across genera as shown with the *Cladosporium fulvum* effector *Avr4* and its homolog in *Mycosphaerella fijiensis*, both of which induce *R*-gene (Cf-4)-mediated resistance responses on tomato (Stergiopoulos et al., 2010). More recently, an *Avr4* homolog from *Dothistroma septosporum* was also shown to trigger a hypersensitive response (HR) when transiently expressed in Cf-4 transgenic *Nicotiana tabacum* (Mesarich et al., 2015).

AvrLm6 is a secreted, proteinaceous effector of *Leptosphaeria maculans*, the causal agent of canola black leg disease, that has been shown to trigger a resistance response on host plants carrying the *Rlm6* resistance gene (Fudal et al., 2007). *AvrLm6* was first identified genetically, with the phenotypic observation of segregation of differential resistance responses of *Brassica napus* and *B. juncea* cultivars to the progeny of *B. juncea*-virulent and -avirulent *L. maculans* isolates (Balesdent et al., 2002). Subsequently, the gene encoding AvrLm6 was identified using map based cloning. Southern blot analyses led to the conclusion that *AvrLm6* homologs were restricted to *L. maculans* (Fudal et al., 2007). Since then, *AvrLm6* homologs have been detected in the whole genome sequences of additional *Leptosphaeria* species. It is likely that these were not identified in the Southern blot analysis by Fudal et al. (2007) due to the low nucleotide sequence identity. Homologs are also found in more distantly related fungi, such as *Colletotrichum* and *Fusarium* species (Grandaubert et al., 2014), as well as in *Venturia* species (Bowen et al., 2011). Nothing is known about the function of AvrLm6 or orthologous proteins in any of these fungi. However, it has been demonstrated that two other *L. maculans* avirulence genes; *AvrLm4-7* (Huang et al., 2006) and *AvrLm1* (Huang et al., 2010) contribute to fungal fitness. Expression of *Avrlm6* is highly up-regulated in *L. maculans* during primary infection of *B. napus*, compared with *in vitro* growth (Fudal et al., 2007; Van de Wouw et al., 2010). Expression levels were recorded to be highest at 7 days post inoculation on canola and then, after falling slightly, are maintained at high levels during stem necrosis. Similar expression patterns are seen in other *L. maculans* effectors *AvrLm1, AvrLm4-7*, and *AvrLm11* (Gout et al., 2006; Parlange et al., 2009; Balesdent et al., 2013). *Avrlm6* has been observed at high frequency among *L. maculans* populations in Europe, in the absence of *Rlm6* resistance (Balesdent et al., 2006; Stachowiak et al., 2006), but when selection pressure from *Rlm6* resistance has been applied in experimental trials, the resistance was overcome after just 3 years (Brun et al., 2000). *AvrLm1* (Gout et al., 2007) and *AvrLm4-7* (Daverdin et al., 2012) alleles have also been reported at high levels in the absence of selection pressure from the cognate resistance genes in the field, but, as seen with *AvrLm6*, virulent alleles increase rapidly once resistance genes are introduced, despite the fitness costs to the pathogens (Huang et al., 2006, 2010).

A diverse array of mechanisms have been shown to generate new virulent *AvrLm6* alleles; including deletion, point mutation, and repeat induced point mutation (RIP; Fudal et al., 2009; Van de Wouw et al., 2010). RIP is a type of mutation, that only occurs in fungi, during sexual crossing and alters duplicated DNA sequences and produces CpA to TpA and TpG to TpA mutations (Cambareri et al., 1989). RIP can also impact sequences neighboring duplicated DNA sequences, such is the case with *AvrLm6* in *L. maculans* where RIP has “leaked” from nearby repetitive DNA sequences into the *Avrlm6* coding sequence (Van de Wouw et al., 2010; Rouxel et al., 2011). *AvrLm6* inhabits a gene poor, AT-rich region of the *L. maculans* genome and is surrounded by large numbers of repetitive sequences comprising predominantly long terminal repeat (LTR) retrotransposons (Pholy, Olly, Polly, and Rolly), which appear to have been RIP-affected (Gout et al., 2006; Fudal et al., 2007).

Research into the *AvrLm6*-like genes from *V*. *inaequalis* (*ALVi*s) and *V. pirina* (*ALVp*s) was conducted to determine a possible role for these SSPs in scab disease development. The predicted secretomes and WGS of a single *V. pirina* and 4 *V. inaequalis* strains were mined for sequences with similarity to *AvrLm6* and their surrounding genomic contexts were examined to further understand the evolution and nature of the gene family expansion. The large size of the gene families of *AvrLm6*-like genes and probable functional redundancy hindered the possibility of gene disruption or silencing to determine function. Timing and location of *AvrLm6*-like gene expression was investigated to further understand the role of these proteins in *Venturia*. An *L. maculans* isolate lacking the avirulent *AvrLm6* allele was also transformed with a *V. inaequalis ALVi* gene to determine whether the phenotype of avirulence on the *Rlm6-*containing *B. napus* could be restored (i.e., by complementation of the heterologous gene) indicating conservation of function.

## Materials and Methods

### *Venturia* Isolates and Genomic Resources used in This Work

The genomic resources used in this study are detailed below in **Table 1**. The *V. inaequalis* Vi1 genome was used to investigate the genomic environment relating to the *ALVi* gene family as it is the genome that is annotated the most completely, and we have transcriptome data for various time points and growth conditions [BioProject (ID PRJNA261633): *in vitro*: SRR1586226, 2 dpi *in planta*: SRR1586224, 7 dpi *in planta*: SRR1586223J] which has been used to inform gene prediction.

**Table 1:** Genome sequence summary statistics for each *Venturia* isolate included in this analysis.

	*Venturia inaequalis*				*V. pirina*
Race	1	1,10	5	Unknown	Unknown
Name	Vi1 (MNH120)	B04	R5	1389	11032
Host	*Malus* × *domestica*	*Malus* × *domestica*	*Malus* × *domestica*	*Eriobotrya japonica*	*Pyrus communis*
Estimated genome coverage	120x	90x	55x	89x	74x
Number of scaffolds	1,012	1,415	1,308	1,040	364
Size (Mb)	55	61	72	62	41
Scaffold N50	233,760	136,376	263622	213,378	332,167
Number of contigs	3,520	3,716	1,689	2,557	1,561
Contig N50	65,081	84,371	92,135	121,114	146,156
BUSCO completeness (% of complete single copy orthologues identified from a reference set)	96.5%	97%	96%	97%	97%
Percentage of genome covered by repetitive elements	4.11%	33.41%	Not determined	35.60%	7.28%


The *V. inaequalis* Vi1 and the *V. pirina* genomes (Cooke et al., 2014) are publicly available via the MycoCosm genome portal at JGI^1^^,^^2^ . All *V. inaequalis* and *V. pirina* isolates used in this study have been reported previously (Stehmann et al., 2001; Le Cam et al., 2002; Win et al., 2003; Bowen et al., 2009; Broggini et al., 2011; Cooke et al., 2014; Caffier et al., 2015). The rest of the genomes were only used to extract the ALVi predicted protein sequences, these are included in the Supplementary Material.

### Identification of AvrLm6 Homologs in Public Sequence Databases

The NCBI (National Centre for Biotechnology Information^3^) non-redundant (nr) public sequence database was queried with the AvrLm6 (GenBank: CAJ90695) predicted protein sequence using PSI-Blast with default settings and an e-value cut-off of 1E-5. Predicted proteins returned from the PSI-Blast search were collected and used in blastp analysis of protein databases at JGI (Joint Genome Institute^4^) and the Fungal Genomes proteins available at the Broad Institute^5^ also using 1E-5 *e*-value cut-off. In order to identify homologs which had not been predicted, tblastn searches were performed, with the same stringency as above.

### Identifying *ALVp* and *ALVi* Gene Families

Two criteria were used to decide which genes to include in the *ALVi* and *ALVp* gene families. Firstly, standalone PSI-Blast (blast + 2.2.29) was used with AvrLm6 (GenBank: CAJ90695) protein sequence as the query with an *e*-value cut-off of 1E-5 against the protein catalogs of each *Venturia* isolate. The predicted proteins identified that met this first criterion were then used in back-blast searches to screen the NCBI (National Centre for Biotechnology Information^3^) nr public sequence database. If the *e*-value relating to the most similar protein was less than, or equal to 1E-2 to AvrLm6 or one of its homologs from another fungal species (defined above) the sequence was considered an ALVi or ALVp. All ALVp and ALVi amino acid sequences are included in the Supplementary Material (Data Sheet 1).

### Phylogenetic Analysis

Predicted amino acid sequences of all *ALVi* and *ALVp* genes from *V. inaequalis* isolate Vi1 and *V. pirina* isolate 11032 were aligned using MUSCLE multiple sequence alignment implemented in the MEGA6 program (Tamura et al., 2013) and a maximum likelihood tree was derived from the alignment using the WAG substitution model tested with bootstrapping (500). Another tree was constructed in an identical manner with the inclusion of amino acid sequences from additional species including; AvrLm6 (GenBank: CAJ90695) and other homologs from; *L. maculans* (GenBank:XP_003843096.1), *Colletotrichum gloeosporioides* (GenBank:XP_007281214.1 and XP_007275717.1), *C. higginsianum* (GenBank:CCF39162.1), *C. obliculare* (GenBank:ENH83850.1 and ENH87246.1) and *Fusarium oxysporum* (GenBank:EXK76127.1) using a WAG substitution model and 1000 bootstrap tests. The trees were drawn with Figtree V1.4.2.

### Quantification of Gene Expression of Selected *ALVis* in *V. inaequalis* Isolate Vi1

Expression analysis of eight *ALVi* genes from Vi1 (*ALVi_Vi1_4, ALVi_Vi1_5*, *ALVi_Vi1_7*, *ALVi_Vi1_9*, *ALVi_Vi1_14*, *ALVi_Vi1_15, ALVi_Vi1_17 and ALVi_Vi1_22*) was carried out by qRT-PCR out on a LightCycler^®^ 480 instrument (Roche) using LightCycler^®^ 480 SYBR Green I Master reagents (Roche). Each 10 μl reaction contained 5 μl of mastermix (2X) 0.5 μl of each primer (at 5 μM) and 4 μl of cDNA (see below). The PCR cycling conditions were as follows; Initial denaturation for 5 min 95°C, followed by 45 cycles of 95°C 10 s, 60°C 10 s, and 72°C 8 s. Primers used in this experiment are detailed in the **Supplementary Table S2**. β-tubulin and the 60 s ribosomal L12 gene were used as reference genes as previously described (Kucheryava et al., 2008). These *ALVi*s were chosen for qRT-PCR analysis based on preliminary RNA-seq data which showed they were up-regulated during infection when compared with growth *in vitro*. Three biological replicates were included for each time point and each biological replicate was triplicated for technical replicates.

RNA used in qRT-PCR experiments was extracted from Vi1 cultures growing on cellophane amended potato dextrose agar (PDA) plates 10 days post inoculation to test *in vitro* gene expression and from detached leaf infections at 3, 7, and 14 days post inoculation (dpi). These infections were performed as previously described (Win et al., 2003). RNA was extracted from all samples following a published protocol (Chang et al., 1993).

To grow apple leaves for the detached leaf assays, apple seeds were sourced from open pollinated *Malus* × *domestica* ‘Royal Gala.’ Surface-sterilized seeds were stratified at 4°C in water-saturated vermiculite for 6–12 weeks to enable germination. Germinated seeds were planted in compost at 21°C with a 12 h light period/day under 4C W SHP-TS lights (Sylvania, Danvers, MA, USA) inoculations were performed between 4 and 6 weeks after planting in compost.

### Localisation of an ALVi Protein during Infection

The *ALVi_Vi1_5* gene from *V. inaequalis* Vi1 was chosen for localisation analysis using fluorescent protein (YFP) tagging. The enhanced yellow fluorescent protein (eYFP) gene had been adapted for *V. inaequalis* codon usage. The *ALVi_Vi1_5* gene was chosen as preliminary RNA-seq data and qRT-PCR analysis showed that its expression was up-regulated during infection, compared with growth *in vitro*. The PJK4 plasmid containing a fusion of ALVi_Vi1_5:eYFP (PJK4: ALVi_Vi1_5:eYFP) was used as a binary vector for *Agrobacterium*-mediated transformation of *V. inaequalis* isolate Vi1 (Fitzgerald et al., 2003). The expression cassette was constructed with overlap extension PCR (Heckman and Pease, 2007), joining the ALVi_Vi1_5 predicted promoter (1003 bp upstream of start codon) and coding sequence in frame with the eYFP gene and the predicted ALVi_Vi1_5 terminator (1001 bp downstream of the stop codon), and cloned into PJK4 at the *Spe*1 and *Bgl*2 cloning sites. The predicted promoter and terminator contained no predicted open reading frames. Primers used in the construction are detailed in the **Supplementary Table S3**. The sequence of the insert was validated by Sanger sequencing (Australian Genome Research Facility).

To observe localization of the fluorescent fusion protein *in planta*, apple hypocotyls were inoculated with 5 μl droplets of spore suspension (1 × 10^5^ spores/ml) prepared from PJK4: ALVi_Vi1_5:eYFP transformants or the wild type, untransformed Vi1 isolate. Infected hypocotyls were incubated at 20°C in darkness for up to 14 days. PDA plates amended with cellophane membranes were also inoculated with the spore suspensions and stored under the same conditions. Infections were observed at 2, 7, and 14 dpi using a Leica TCS SP2 Confocal Microscope (Leica Wetzlar Germany) using the 100× magnification, oil immersion lens. Hypocotyls were grown from *Malus* × *domestica* ‘Royal Gala’ seed as described above for detached leaf assay.

### Complementation Assays using *ALVi-Vi1_8* Transformed into an *L. maculans* Virulent Isolate

A complementation assay was carried out to see if *L. maculans* expressing *ALVi_Vi1_8* driven by the *AvrLm6* promoter could confer avirulence toward the *Rlm6*-containing *B. napus* cultivar ‘Aurea’ which is resistant to *L. maculans* isolates expressing the *AvrLm6* gene. *ALVi_Vi1_8* was chosen because it had the most similar predicted sequence to AvrLm6 based on the blastp analysis (32% identity). The complementation cassette contained *ALVi_Vi1_8* (477 bp) flanked by regions upstream (955 bp) and downstream (1255 bp) of *AvrLm6* amplified from *L. maculans* isolate v23.1.3. This fragment was constructed using overlap extension PCR (Heckman and Pease, 2007) and cloned into the binary vector pZP-Nat containing the *nourseothricin acetyltransferase* gene (Elliott and Howlett, 2006). *Agrobacterium*-mediated transformation (Gardiner and Howlett, 2004) was used to transform *L. maculans* isolate M1, which lacks the *AvrLm6* gene, with the construct. Pathogenicity testing were performed on cotyledons of *B. napus* ‘Westar’(lacking *Rlm6*) and *B. juncea* ‘Aurea’ (carrying *Rlm6*) as previously described (Van de Wouw et al., 2014). Expression of the transgene during infection was confirmed by RT-PCR. Primers used in vector construction and RT-PCR are listed in the **Supplementary Table S4**.

### Genomic Context, RIP, and Repeat Regions

The prediction suite REPET 2.2 (Flutre et al., 2011) was used for the detection and annotation of transposable elements (TEs). RipCrawl^6^ was used to predict areas of the genome that had undergone RIP based on composite RIP indices (CRI) with the same restraints that have previously been reported (de Wit et al., 2012). Bedtools (Quinlan and Hall, 2010) was used to calculate distances from *ALVi* genes to features of interest. The above analysis was also done on a set of 439 core eukaryotic reference genes identified using the NCBI eukaryotic clusters of orthologous groups (KOGs). The KOG numbers and the corresponding gene names in *V. inaequalis* isolate Vi1 are listed in the **Supplementary Table S5**.

## Results

### *AvrLm6* Homologs are Found Across Multiple Fungal Genera and Form Expanded Gene Families in *V. pirina* and *V. inaequalis*

**FIGURE 1:**
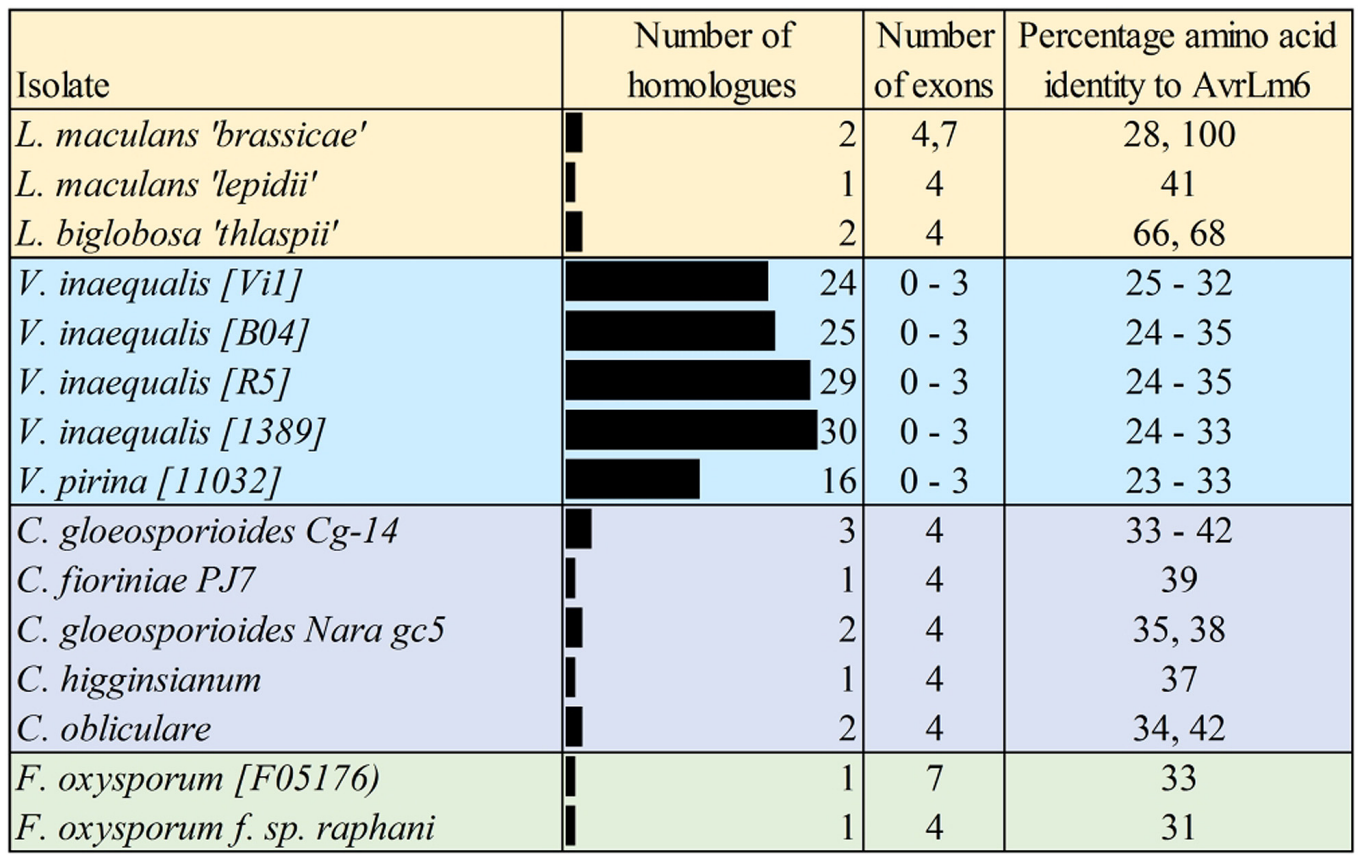
**Summary of the *AvrLm6* homologs identified in public and private sequence databases.** The number of exons predicted for each gene are shown as well as the percentage amino acid identity to the AvrLm6 predicted protein (CAJ90695) from blastp searches. Accession numbers and further details of homologs are detailed in the **Supplementary Table S1**.

**FIGURE 2:**
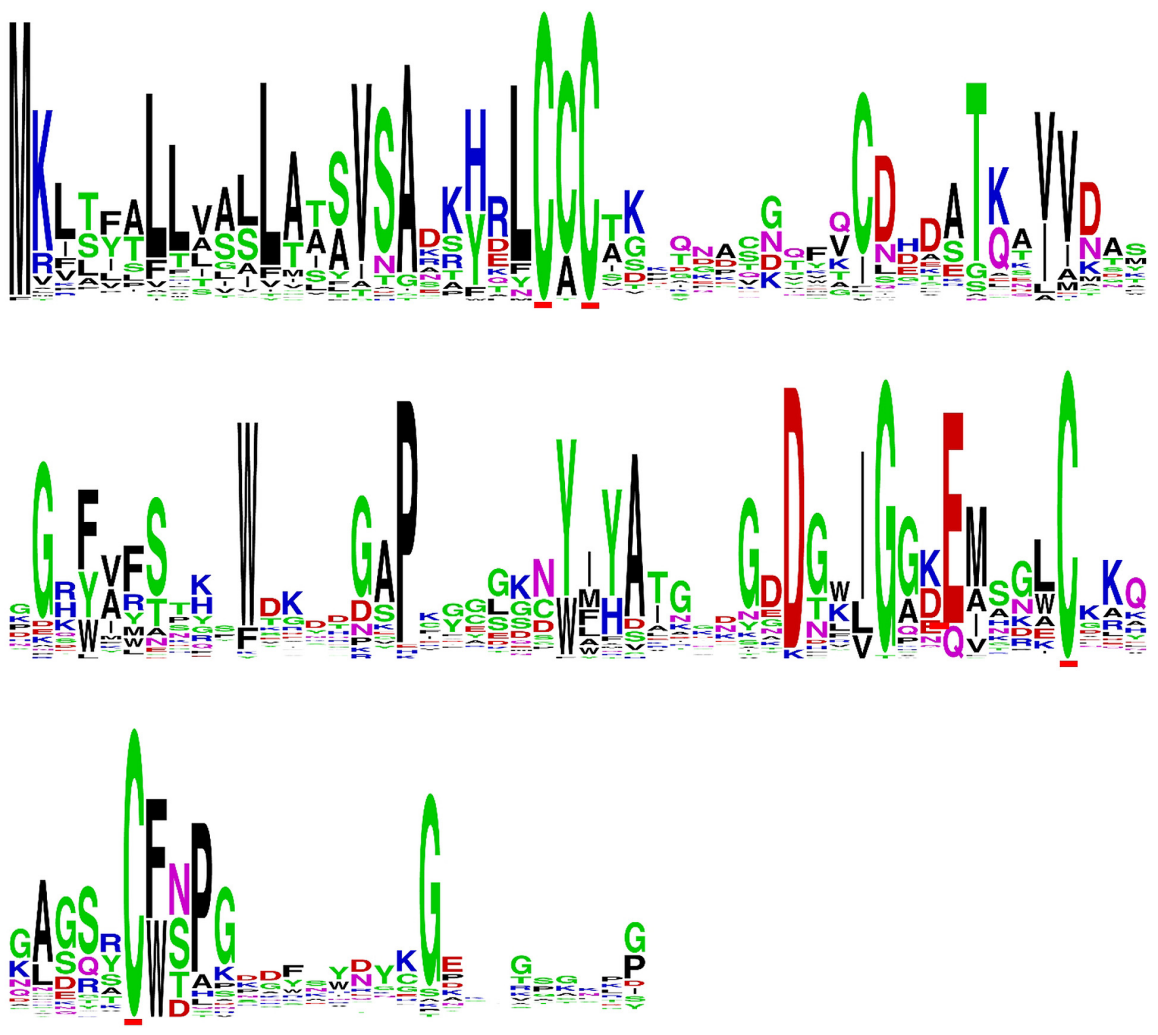
**Sequence logo of a multiple sequence alignment of predicted protein sequences including AvrLm6 and a paralogue from *Leptosphaeria maculans* (GenBank:CAJ90695 and XP_003843096.1) homologs from *Colletotrichum gloeosporioides* (GenBank:XP_007281214 and XP_007275717.1), *Fusarium oxysporum* (GenBank:EXK76127.1), *C. higginsianum* (GenBank:CCF39162.1), *C. fiorinae* (GenBank:XP_007599415.1) as well as ALVI_1 to ALVi_24 from *Venturia inaequalis* Vi1 and ALVp_1 to ALVp_16 from *V. pirina* isolate 11032**.

In addition to the *AvrLm6* homologs that were previously reported in the genomes of *C. higginsianum*, *C. gloeosporioides*, *F. oxysporum*, and *L. biglobosa* (Grandaubert et al., 2014), we report additional *AvrLm6* homologs in *C. orbiculare, C. fioriniae*, and *F. oxysporum* f. sp. *raphani* (**Figure 1**). We observed differences in the copy number of homologs identified in each genome which varied, even within species (**Figure 1**). *Venturia* genomes had up to 30 copies, whereas no other genera had more than 3. In the case of *F. oxysporum* and *Leptosphaeria* species there are also isolates which appear to have no homologs. When all ALVi and ALVp predicted protein sequences were used as blast queries of the NCBI nr database, the levels of sequence identity to AvrLm6 homologs ranged from 24 to 41%. Only two sequences returned the *L. maculans* AvrLm6 protein as the most similar (ALVp_11032_13 from *V. pirina* isolate 11032 and ALVi_1389_1 from Vi1389). All other *V. pirina* and *V. inaequalis* homologs were more similar to AvrLm6 homologs from either *Fusarium* or *Colletotrichum* species than to AvrLm6, and none had similarity to any other *L. maculans* proteins other than to AvrLm6 or related homologs. The multiple sequence alignment revealed four cysteine residues that are conserved in all homologs (**Figure 2**), despite low sequence similarity overall. *ALVis* and *ALVps* also have different gene structure when compared to the homologs found in all the other species, which are generally well conserved with four exons in all homologs except EGU73747.1 from *F. oxysporum* Fo5176 and XP_003843096.1 from *L. maculans* which both have seven exons (**Figure 1**). The gene structures of *ALVp*s and *ALVi*s vary, in that they either have no introns, one intron in the five prime un-translated region (5′ UTR), or either one or two introns in the coding sequence of the gene. The most common structure found in the *ALVi*s is a single intron in the 5′ UTR present in 19 of the 24 *ALVi*s identified in Vi1. We were able to confirm these predicted gene structures in 11 of the 24 genes with reference to mapped RNA-seq reads. All of the AvrLm6 homologs identified in public databases, (with the exception of *F. oxysporum* EGU73747.1) and all of the ALVi and ALVp members we have described here, are predicted to contain a signal peptide sequence at their N terminus and so are expected to be secreted by the classical secretory pathway.

### Expansion of *ALVp* and *ALVi* Gene Families

**FIGURE 3:**
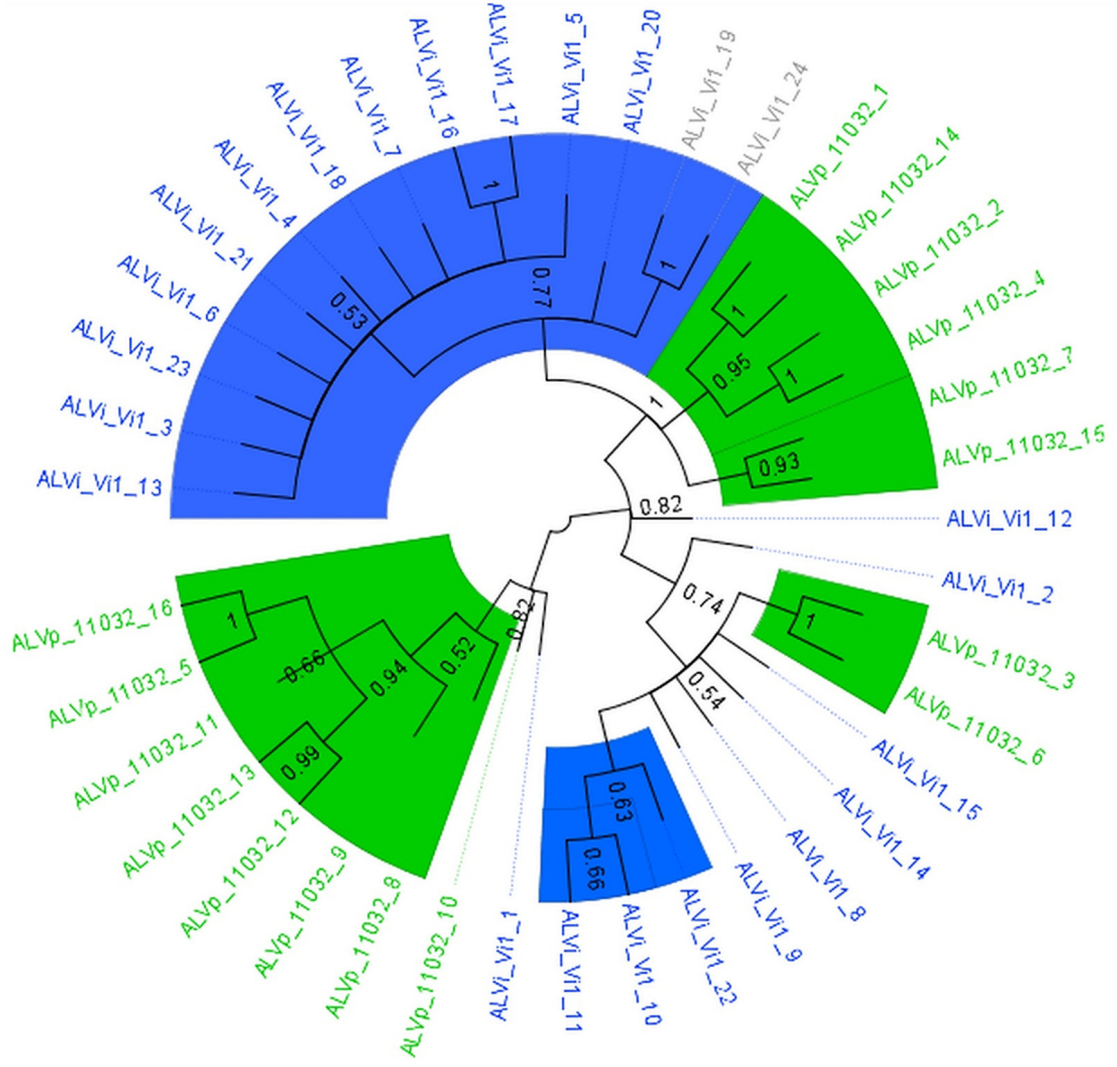
**Maximum likelihood tree calculated on multiple sequence alignment of ALVi and ALVp predicted protein sequences from *V. inaequalis* isolate Vi1 and *V. pirina* isolate 11032 using a WAG substitution model and 500 bootstrap tests.** Species-specific clades with over 75% bootstrap support are highlighted with colored wedges. Sequence names in green are from *V. pirina* isolate 11032 and blue from *V. inaequalis* Vi1. Gray sequence labels represent Vi1 genes for which we have no evidence for gene expression from in planta or *in vitro* growth.

**FIGURE 4:**
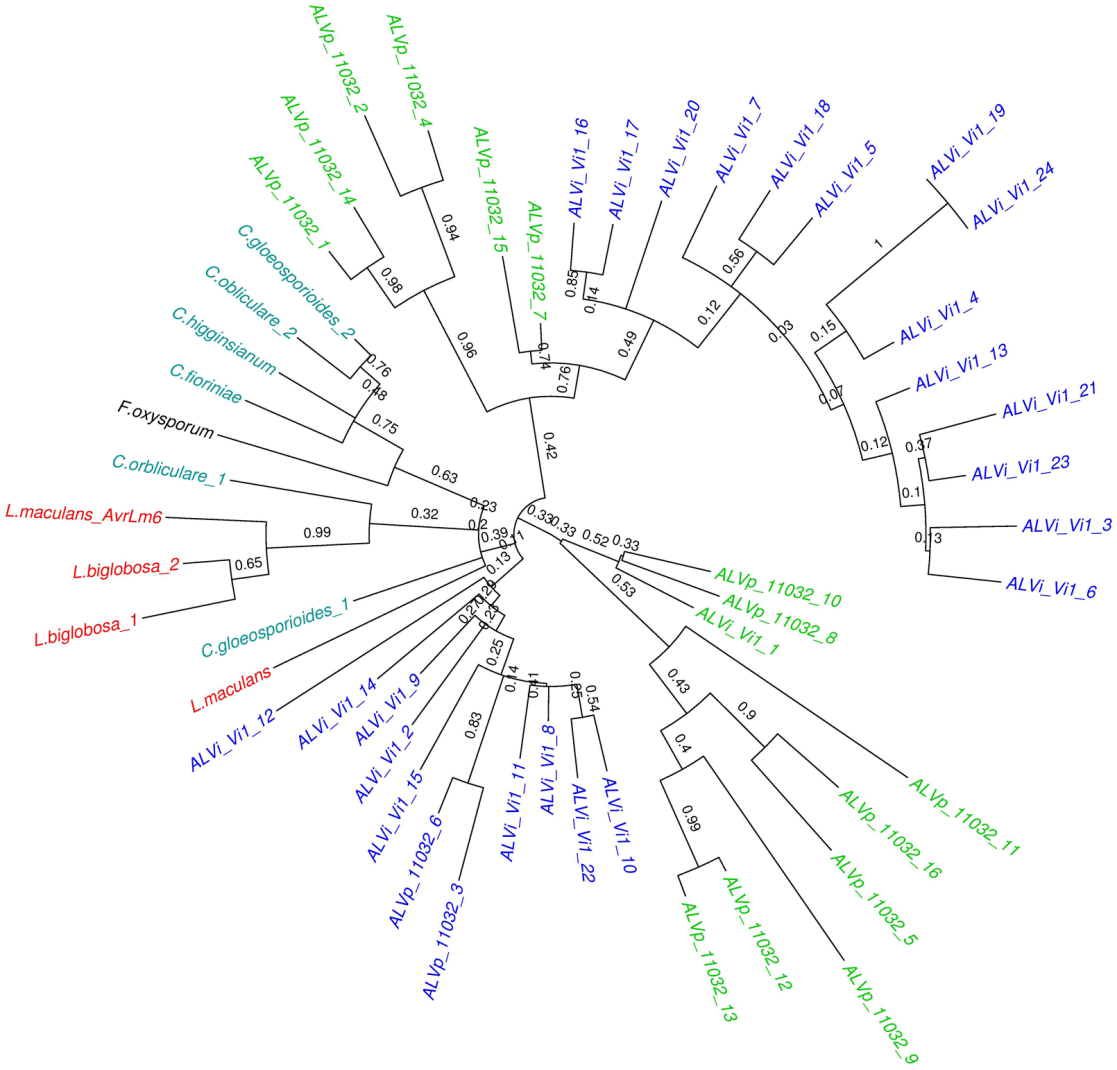
**Maximum likelihood tree calculated on multiple sequence alignment of all ALVi and ALVp predicted protein sequences from *V. inaequalis* isolate Vi1 and *V. pirina* isolate 11032 as well as AvrLm6 (GenBank: CAJ90695) and a paralogue from *L. maculans* and homologs from *C. gloeosporioides* (GenBank:XP_007281214.1 and XP_007275717.1), *C. higginsianum* (CCF39162.1), *C. orbliculare* (ENH83850.1) and (ENH87246.1) and *F. oxysporum* (EXK76127.1) using a WAG substitution model and 1000 bootstrap tests.** Sequence names in green are from *V. pirina* isolate 11032 and blue from *V. inaequalis* Vi1, red are from *Leptosphaeria* species, teal are *Colletotrichum* species, and black is *Fusarium oxysporum*.

The maximum likelihood tree derived from the multiple sequence alignment of the ALVi and ALVp amino acid sequences (**Figure 3**) formed four strongly supported clades with 95, 93, 100, and 94% support that included only ALVp sequences, and one large clade, with 77% support, containing only ALVi sequences. Mixed clades containing sequences from both species did not have strong statistical support (**Figure 3**). When AvrLm6 from *L. maculans* and orthologues from *Fusarium* and *Colletotrichum* species are included in the tree, there were many clades with low support (**Figure 4**) making it difficult to resolve the evolutionary relationship between *ALVi* and *ALVp* families with orthologues in those species, however, a two well supported clades of ALVp and ALVi predicted proteins can be resolved supporting the recent expansion of the gene family in *Venturia* sp. AvrLm6 clustered in a strongly supported clade with the homologs from *L. biglobosa*, but there was not strong statistical support for clustering between these *Leptosphaeria* predicted proteins and those of any other species.

### *ALVis* are Found in Gene Poor Regions Associated with Transposable Elements

**FIGURE 5:**
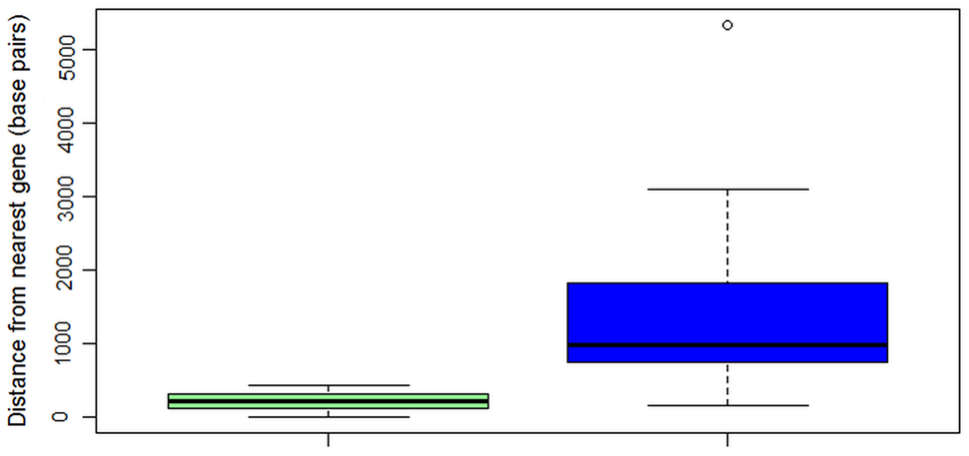
**Box and whisker plot showing distances in base pairs from nearest gene to core eukaryotic genes (*n* = 439, green), or to *ALVi* genes (*n* = 24, blue) in *V. inaequalis* Vi1**.

*ALVi* genes in the Vi1 genome are found in gene poor regions (**Figure 5**) when compared with a reference set of 439 core eukaryotic genes (**Supplementary Table S5**). The mean distance to the nearest gene was 1,370 bp for the ALVi-coding loci and 484 bp for the core eukaryotic genes, the difference between the means was found to be statistically significant by a Student’s *t*-test (*p*-value < 0.001). *ALVi* sequences are found in close proximity to TEs predicted with the REPET 2.2 pipeline. The mean distance to the nearest TE from an ALVi gene in isolate Vi1 (203 bp) was significantly less (Student’s *t*-test *p*-value < 0.00001), than that of the reference set of core genes (5,882 bp). The most common class of TE associated with the *ALVi* genes was the large retrotransposon derivative (LARD) class, 10 of these occurred within 260 bp of an *ALVi* gene and of those, six overlapped *ALVi*-coding loci. Terminal-repeat retrotransposons in miniature (TRIMs) were the second most common element identified at *ALVi* loci, overlapping with eight *ALVi*-coding sequences. Using the CRI index criteria that has been previously defined (de Wit et al., 2012) we could not find evidence of RIP in any of the *ALVi* coding sequences in isolate Vi1. However, evidence of RIP was found throughout the genome, with 72% of the TEs predicted to be RIP-affected. None of the TEs overlapping the *ALVi* sequences were shown to be RIP affected, but seven *ALVis* loci had evidence of RIP in the nearest neighboring repetitive elements. These elements included two predicted TRIMs, one LARD, as well as fragments of; long terminal repeats (x2) a terminal inverted repeat and a long interspersed nuclear element.

### ALVi_Vi1_5 Localizes to the Stroma during Infection

**FIGURE 6:**
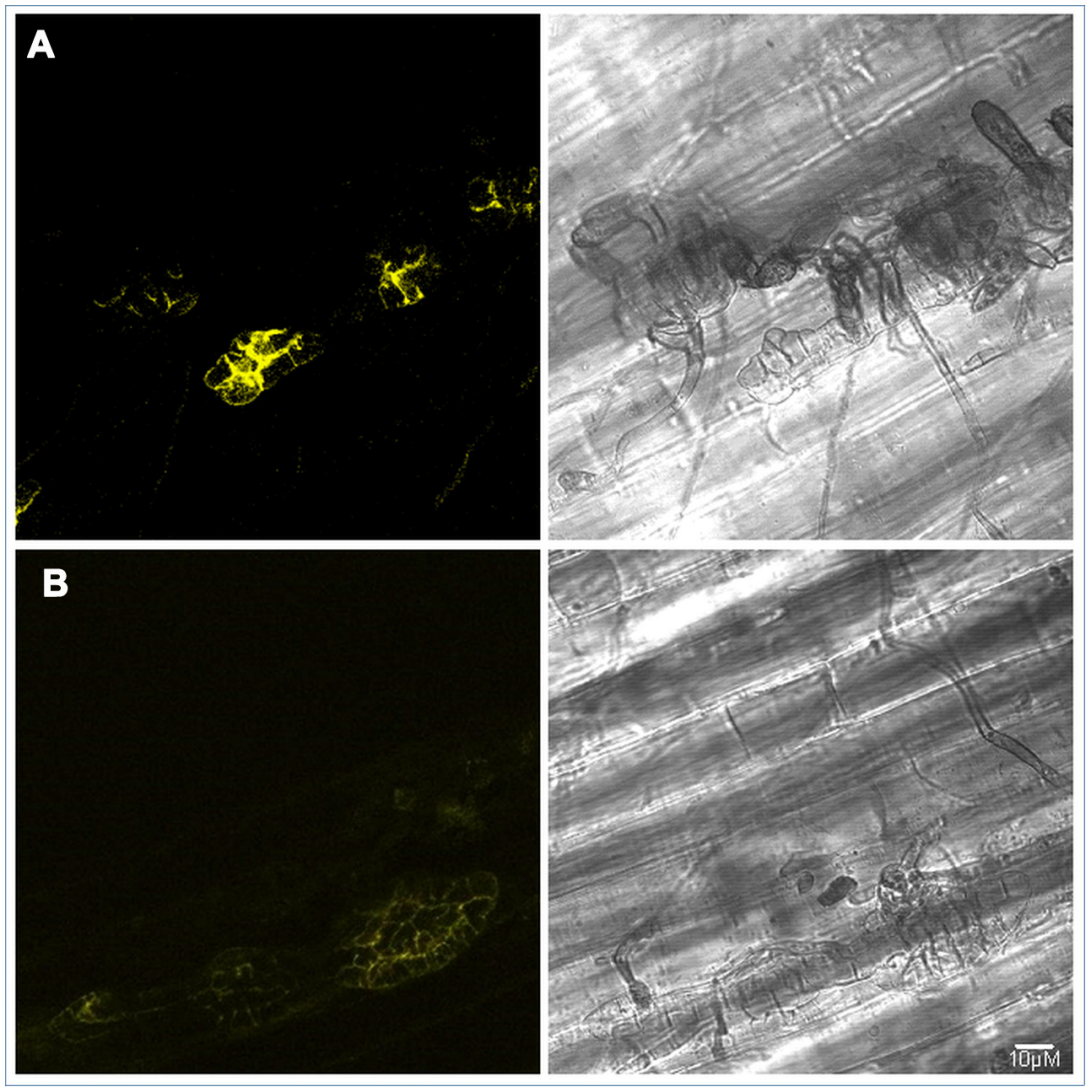
**PJK4:ALVi_Vi1_5: eYFP transformed isolate *V. inaequalis* Vi1 growing on apple hypocotyl viewed with bright field microscopy (right panels) and confocal fluorescent (left panels) 7 days post inoculation (A) and 14 days post inoculation (B).** YFP fluorescence observed in multicellular, sub-cuticular stroma only. Fluorescent images are z stacks.

Fluorescent signal from the ALVi_Vi1_5 fusion protein was visible at 7 and 14 dpi in infected apple hypocotyls (**Figure 6**) but not visible at the earlier infection time points or at any time-points *in vitro.* The signal appeared to localize to the periphery of the sub-cuticular stroma and was not visible in surface hyphae, conidiophores or spores. The similar levels of fluorescence at seven and 14 dpi reflected the qRT-PCR data (**Supplementary Figure S2**) which showed that expression was similar at these time points and higher than that observed at 3 dpi.

### ALVi_Vi_1_8 does not Trigger Rlm6-mediated Resistance in Canola

Expression of ALVi*_Vi1_8* was confirmed by RT-PCR in the *L. maculans* (Isolate M1) transformants containing the complementation vector (**Supplementary Figure S2**), but none of these transformants triggered a resistance response in *B. juncea* ‘Aurea,’ expressing the *Rlm6* resistance gene (**Supplementary Figure S3**). The expected resistance response was observed when *B. juncea* ‘Aurea’ was infected with *L. maculans* (Isolate M1) transformed with the *AvrLm6* avirulence gene. The pathogenicity of the ALVi*_Vi1_8* transformed isolates on the susceptible *B. napus* ‘Westar’ cultivar was indistinguishable from the wild type *L. maculans* expressing the native *AvrLm6* gene and the wild type *L. maculans* M1 isolate.

## Discussion

Fungal effector proteins have long been characterized by their lineage-specific distribution, and were generally thought to be conserved at the species or race level. A rapid rise in the availability of plant pathogen whole genome sequences, especially for those classified within the Dothideomycetes and the Sordariomycetes, has enabled this dogma to be tested by sequence similarity searches. In this way, new homologs of the *L. maculans* effector, AvrLm6, have been identified. The taxonomic distribution of AvrLm6 orthologues is interesting, because while they are found across two different classes of fungi, the Dothideomycetes and the Sordariomycetes, they have apparently only been conserved in a few species within these classes. Furthermore, AvrLm6 from *L. maculans* has higher sequence similarity to orthologues in the Sordariomycetes class than to orthologues from the more closely related *Venturia* species (**Supplementary Figure S1**). One possible explanation of the unusual taxonomic distribution of these genes could be horizontal gene transfer (HGT). There are now many examples of HGT in fungal and oomycete plant pathogens (Soanes and Richards, 2014). The most compelling evidence of HGT is *ToxA*, found in two wheat pathogens. The *ToxA* gene in *Parastagonospora nodorum* appears to have been horizontally transferred from *Pyrenophora tritici-repentis*. The *ToxA* genes in both species had very high nucleotide sequence identity (99.7%) which did not concur with the phylogenetic relationship inferred when comparing the ITS regions and glyceraldehyde-3-phosphate genes (83 and 80% respectively; Friesen et al., 2006). In the case of *AvrLm6* though, the homologs have a high level of sequence divergence and while *AvrLm6* did cluster with the *Colletotrichum* homologs in the phylogenetic tree, this relationship was not supported with bootstrapping. Even within the same species, the presence of these genes is not conserved, for example, we could only identify homologs in two isolates of *F. oxysporum* with blast searches, despite the 25 searchable genomes in the NCBI database. This finding is not simply due to poor gene models in the WGS of fungi as six frame similarity searches also did not reveal orthologues. The *AvrLm6* allele is also highly polymorphic at the population level and can also be absent in some *L. maculans* isolates (Van de Wouw et al., 2010). The deletion of avirulence effector loci is one way that pathogens avoid recognition by resistant plants. The function of AvrLm6 is unknown, however, it is not essential for pathogenicity in *L. maculans.* This may be why it is so poorly conserved in the Dothideomycetes in general. The expansion of the *AvrLm6*-like genes in *Venturia* sp. is clearly against the trend in this group.

In *V. inaequalis* and *V. pirina*, copy number of *AvrLm6*-like genes varies between isolates and species. The *ALVi* and *ALVp* gene family expansions appear to have occurred independently after speciation. This can be seen on the gene tree (**Figure 3**), where strongly supported clades are composed of clusters of paralogues rather than orthologues. *V. inaequalis* and *V. pirina* species are restricted to and separated spatially on different host ranges, but otherwise have very similar lifestyles and biology, hence, differences in the evolution of these gene families may reflect adaptation to a new host.

The mechanism/s responsible for the expansion of the *ALVp* and *ALVi* gene families remains unclear. However, the association between *ALVis* and TEs, predominantly LARDs and TRIMs, suggests that the multiple gene duplications could have been mediated by these elements. Indeed the expansion of the *AVRk1* effector gene family in *Blumeria graminis* has been hypothesized to be driven by the association with LINE1 retrotransposons (Sacristán et al., 2009).

Large retrotransposon derivatives are non-autonomous long terminal repeat (LTR) retrotransposons which have been described in *Triticeae* (Kalendar et al., 2004), rice (Nagaki et al., 2005; Vitte et al., 2007) and fungi (Labbé et al., 2012; Pereira et al., 2015); they consist of LTRs flanking a large non-coding internal domain, and lack the coding domains found in other LTR retrotransposons, which are responsible for self-replication (Kalendar et al., 2004). TRIMs, like LARDs, are non-autonomous retrotransposons, but differ structurally from LARDs, in that they have much shorter internal domains and terminal direct repeats (Witte et al., 2001). TRIMs have been observed within coding and non-coding regions of plant (Witte et al., 2001; Sampath and Yang, 2014) and animal genes (Zhou and Cahan, 2012). It has been observed that TRIMs can be involved in the transduction of host genes, a situation where a host gene or part of host gene becomes part of the replicating transposon, and can lead to gene duplication (Witte et al., 2001). A TRIM identified in *Arabidopsis thaliana* (*Katydid*-At1) was found to contain an open reading frame very similar to the host gene coding for the mRNA decay (NMD) trans-acting factor, as none of the intronic sequences were found in the transduced gene. It was hypothesized that this even occurred by the recombination of the host gene transcript and the TRIM (Witte et al., 2001). Such a method of gene family evolution could account for the variability in gene structure and expansion of the *ALVps* and *ALVis* in *Venturia*. Another explanation is that part of the TRIM may have been recruited by the *ALVi* genes and expansion occurred by another mechanism. The genomic context of the *ALVi*s is similar to that of *Avrlm6*. *AvrLm6* in the *L. maculans* genome (strain v23.1.3) is situated in a gene-poor and AT-rich isochore, adjacent to an abundance of repetitive elements and remnants of transposons (Fudal et al., 2007), however, TRIMs have not been reported in *L. maculans*. Gene sparse regions are a feature of some filamentous plant pathogens and can be niches for effector genes and their evolution (Raffaele and Kamoun, 2012). Despite sharing a similar genomic context to *AvrLm6*, RIP was not detected in any of the *ALVi* sequences in *V. inaequalis.* RIP was detected bioinformatically in other regions of the genome and the majority of TEs were predicted to be affected by RIP, so it appears that RIP machinery is or has been active in the *V. inaequalis* genome. It remains unclear how the *ALVi* gene family was able to expand without being affected by RIP. However, RIP has not been investigated experimentally in *Venturia* sp, so the constraints of the RIP machinery are unknown. It has been shown experimentally in *Neurospora crassa* that duplicate nucleotide sequences of less than 380 were not subject to RIP (Watters et al., 1999). While most *ALVi* sequences are longer than this, it is possible that the *V. inaequalis* RIP machinery has other constraints that preclude the *ALVi* genes from being targets for RIP.

*Venturia* species, upon infection, rapidly form stroma (multicellular, laterally dividing tissue) in the cuticle and subcuticular space (Bowen et al., 2011). It is thought that the stroma is the main feeding and conidia-producing structure in *Venturia*. The *AvrLm6*-like homolog, ALVi_VI1_5 was localized, as a fusion protein with eYFP, to the periphery of the subcuticular stroma cells during infection. This is consistent with the gene expression data with regards to the timing of appearance of stroma during infection. All of the ALVis and ALVps are predicted to be secreted by the classical secretory pathway, however, we still do not know the function of this protein or whether it is released into the apoplast to interface with the plant.

The ALVi_Vi1_8 protein failed to trigger a resistance response in canola giving no indication as to whether the protein behaves in a similar way to AvrLm6. *R* genes have been described previously which recognize interspecific effectors with relatively low sequence similarity; for example the Cf-4–mediated HR in tomato can be triggered by the Avr4 protein from *C. fulvum* as well as homologs from phylogenetically related species; the MfAvr4 protein from *M. fijiensis* which only share 42% amino acid identity (Stergiopoulos et al., 2010) and DsAvr4 from *D. septosporum* which shares 51.7% identity (de Wit et al., 2012). It has also been frequently demonstrated, however, that small variations in effector sequence can abolish avirulence functionality. Such is the case with the Cf-4 mediated resistance described above, which can be disrupted by exchanging a single conserved proline residue (Mesarich et al., 2015). An *AvrLm6* allele with a single amino acid substitution has also been found in Rlm6-virulent isolates collected from the field (Van de Wouw et al., 2010).

The ALVp and ALVi predicted proteins have typical characteristics of effectors; i.e., they are all small, predicted to be secreted, cysteine rich, contain no known functional domains, and many are upregulated during infection compared to *in vitro* growth. Furthermore, these expanded protein families share sequence similarity to an avirulence effector, known to trigger a specific resistance response in canola to *L. maculans*. The variability in copy number and sequence divergence of ALVis and ALVps between different species and isolates of *Venturia*, also make these proteins strong candidates for effectors (possibly host specificity determinants). Elucidating the roles of the *Venturia* ALVp and ALVi proteins will be challenging, as there is likely to be a degree of functional redundancy among them. The availability of the whole genome and transcriptome sequences for these fungi will facilitate the determination of the genetic basis of physiological races, governing host and cultivar specificity in *Venturia*. This in turn will assist plant breeding efforts to select for more durable resistance against scab diseases.

## Conflict of Interest Statement

The authors declare that the research was conducted in the absence of any commercial or financial relationships that could be construed as a potential conflict of interest.

## Footnotes

1 http://genome.jgi.doe.gov/Venin1/Venin1.home.html

2 http://genome.jgi.doe.gov/Venpi1/Venpi1.home.html

3 http://www.ncbi.nlm.nih.gov/

4 http://www.jgi.doe.gov/

5 http://www.broad.mit.edu/

6 https://bitbucket.org/arobinson/sciencescripts
