# Supplementary material for: A Large Family of AvrLm6-like Genes in the Apple and Pear Scab Pathogens, Venturia inaequalis and Venturia pirina
Source: Front Plant Sci. 2015 Nov 17;6:980. doi: 10.3389/fpls.2015.00980 (PMC4646964; doi:10.3389/fpls.2015.00980)
Supplement: Supplementary file 2 [file Data_Sheet_2.DOCX]

Supplementary Material

**A large family of *AvrLm6*-like genes in the apple and pear scab pathogens,** ***Venturia inaequalis* and *Venturia pirina***

**Jason B. Shiller^1^, Angela van de Wouw^2^, Adam P. Taranto^1^, Joanna K. Bowen^3^, David Dubois^2^, Andrew J. Robinson^1,4^, Cecilia H. Deng^3^, Kim M. Plummer^1*^**

*** Correspondence:** Kim Plummer: K.Plummer@latrobe.edu.au

**Table S1.** AvrLm6 homologues identified at the NCBI (National Centre for Biotechnology Information; <http://www.ncbi.nlm.nih.gov/>) non-redundant (nr) public sequence database from blastp analysis using AvrLm6 (CAJ90695.1) predicted protein as a query.

| **Genbank Accession** | **Description** | **E value from blastp** | **Percentage amino acid identity** |
| --- | --- | --- | --- |
| XP_007281214.1 | m6 protein [*Colletotrichum gloeosporioides* Nara gc5] | 2.00E-17 | 38% |
| EQB45226.1 | hypothetical protein CGLO_15926 [*Colletotrichum gloeosporioides* Cg-14] | 3.00E-17 | 42% |
| EQB59119.1 | hypothetical protein CGLO_00533 [*Colletotrichum gloeosporioides* Cg-14] | 6.00E-15 | 37% |
| EXK76127.1 | hypothetical protein FOQG_19113 [*Fusarium oxysporum* f. sp. *raphani* 54005] | 3.00E-14 | 31% |
| CCF39162.1 | hypothetical protein CH063_00307 [*Colletotrichum higginsianum*] | 1.00E-13 | 37% |
| EGU73747.1 | hypothetical protein FOXB_15741 [*Fusarium oxysporum* Fo5176] | 1.00E-13 | 33% |
| ENH83850.1 | m6 protein [*Colletotrichum orbiculare* MAFF 240422] | 6.00E-11 | 42% |
| XP_007599415.1 | hypothetical protein CFIO01_09995 [*Colletotrichum fioriniae* PJ7] | 2.00E-10 | 39% |
| XP_007275717.1 | m6 protein [*Colletotrichum gloeosporioides* Nara gc5] | 2.00E-09 | 35% |
| EQB43347.1 | hypothetical protein CGLO_18005 [*Colletotrichum gloeosporioides* Cg-14] | 2.00E-08 | 33% |
| ENH87246.1 | m6 protein [*Colletotrichum orbiculare* MAFF 240422] | 1.00E-07 | 34% |
| XP_003843096.1 | predicted protein [*Leptosphaeria maculans* JN3] | 7.00E-07 | 28% |
|  |  |  |  |

**Table S2**. Primers used in the RT-qPCR analyses

| **Gene** | **Forward (5’-3’)** | **Reverse (5’-3’)** |
| --- | --- | --- |
| *ALVi_Vi1_9* | ATTTGCCGTTGCTGGTGT | TCATACTCGCTTCTCGTTGC |
| *ALVi_Vi1_15* | ACCCTGACTCTGCCCAAAA | GGCGTAAATCCAACCATCC |
| *ALVi_Vi1_7* | TCAACCTCACATACCGTCCA | CCGACCCGTCATATTTCAGT |
| *ALVi_Vi1_17* | AGTTCACGTTCACCCTCCTC | AATCTGAGCCTTCCTGATGTTT |
| *ALVi_Vi1_5* | GATTGGGTGGAAAGAAATGAAG | AGATCGCCCTTGTAGTCGTG |
| *ALVi_Vi1_22* | CGAAATACCCGTTCACTTGG | GGTCCTTATCCCCATTCGTT |
| *ALVi_Vi1_14* | AACTGGGACTGGGGCAAA | CAATGGGTATCGTCTTCTCCA |
| *ALVi_Vi1_4* | CAGTTCGCATTCACCAAACA | TCATTTCCTTCTGCCCAATC |

**Table S3**. Primers used in construction of the *ALVi_Vi1_5:eYFP* vector

| **Amplicon** | **Forward (5’-3’)** | **Reverse (5’-3’)** |
| --- | --- | --- |
| ALVi_Vi1_5_CDS and promoter | GAGAGAACTAGTAGGAGATTGAACATCTACCTA | CCTTG GAGAC ACCTG TAAGT GATGG AATCC |
| YFP | ACTTACAGGTGTCTCCAAGGGTGAGGAGCT | CTATTCCTTTCTACTTGTAGAGCTCGTCCA |
| ALVi_Vi_5 terminator | CTACAAGTAGAAAGGAATAGGGGACAACAT | GAGAGAAGATCTGAAGGTTGGTGCTACAAGT |

**Table S4**. Primers used in construction of pZP-Nat:ALVi_Vi1_8 complementation vector

| **Amplicon** | **Forward Primer (5’-3’)** | **Reverse Primer (5’-3’)** |
| --- | --- | --- |
| *AvrLm6* downstream fragment) | GCTTTTGGAGTTGGTCATGGCTATT | GGCGTCTAGATTATCGACGAACGGAGCAC |
| *AvrLm6* promoter | TATAGGATATCC AATCGGCAGCTTGATACGAG | AGCTTCATGCTAGGTTATAAATAGG |
| *ALVi_Vi1_21* coding sequence | TATTTATAACCTAGCATGAAGCTCTTATAC | TGGAGTTGGTCATGGCTATTCAGAAGGACA |
| *Actin* | TTGGTCTTGAAAGCGGTGGTAT | CATCACTGTCCCACGAATTG |

Figure S1. Relative transcript compared with β-tubulin and the 60s ribosomal reference genes abundance as determined by qRT-PCR of *ALVi* genes from isolate Vi1 under different growth conditions. Error bars indicate standard error over three biological replicates. In vitro growth on cellophane amended PDA plates = blue. Detached leaf assays at 3 d.p.i (orange), 7 d.p.i (grey) and 14 d.p.i (yellow)


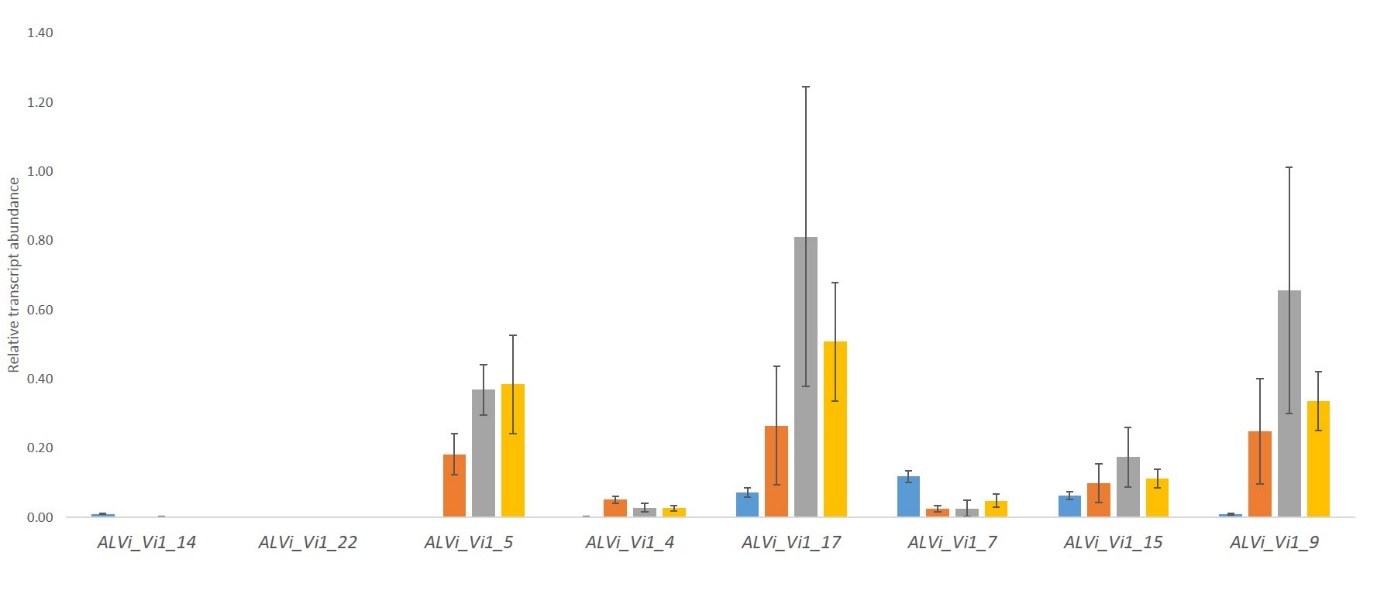


Figure S2. Expression of the *ALVi_Vi_8* gene from *V. inaequalis* in *L. maculans* transformants. The top panel shows amplified portion of the actin cDNA from *L. maculans;* the lower panel the *ALVi_Vi1_21* cDNA amplicon. Lanes; 1. M1 (+RT), 2. M1 (-RT), 3. M1+AvrLm6 (+RT), 4. M1+AvrLm6 (-RT), 5. M1+ALVi_Vi_1_8 #2 (+RT), 6. M1+ALVi_Vi_1_8 #2 (-RT), 7. M1+ALVi_Vi_1_8 #5 (+RT), 8. M1+ALVi_Vi_1_8 #5 (-RT), 9. M1+ALVi_Vi_1_8 #7 (+RT), 10. M1+ALVi_Vi_1_8 #7 (-RT), 11. M1+ALVi_Vi_1_8 #11 (+RT), 12. M1+ALVi_Vi_1_8 #11 (-RT), 13. M1+ALVi_Vi_1_8 #5 gDNA 14. Negative control, no template.


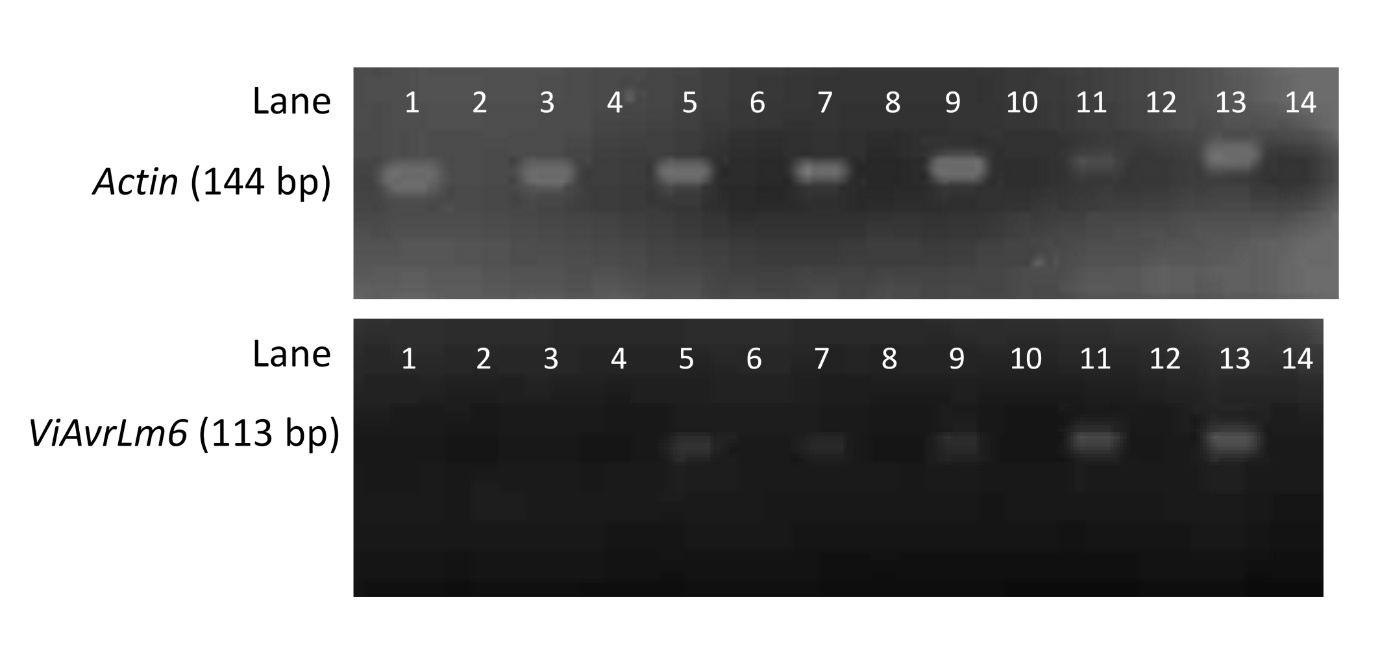


Figure S3 Pathogenicity tests showed that *Leptosphaeria maculans* isolates expressing the *ALVi_Vi1_8* gene did not trigger a resistant, hypersensitive response in ‘Aurea’ canola carrying the *Rlm6* resistance gene but caused similar disease symptoms as seen on Westar canola lacking the *Rlm6* *R* gene. Left panel controls: wild type *L.* *maculans* M1 isolate and one M1 isolate transformed with *AvrLm6*; right panel four *L. maculans* M1 isolates (#2, #5, #7 and #11) transformed with the *ALVI_Vi1_8* gene.


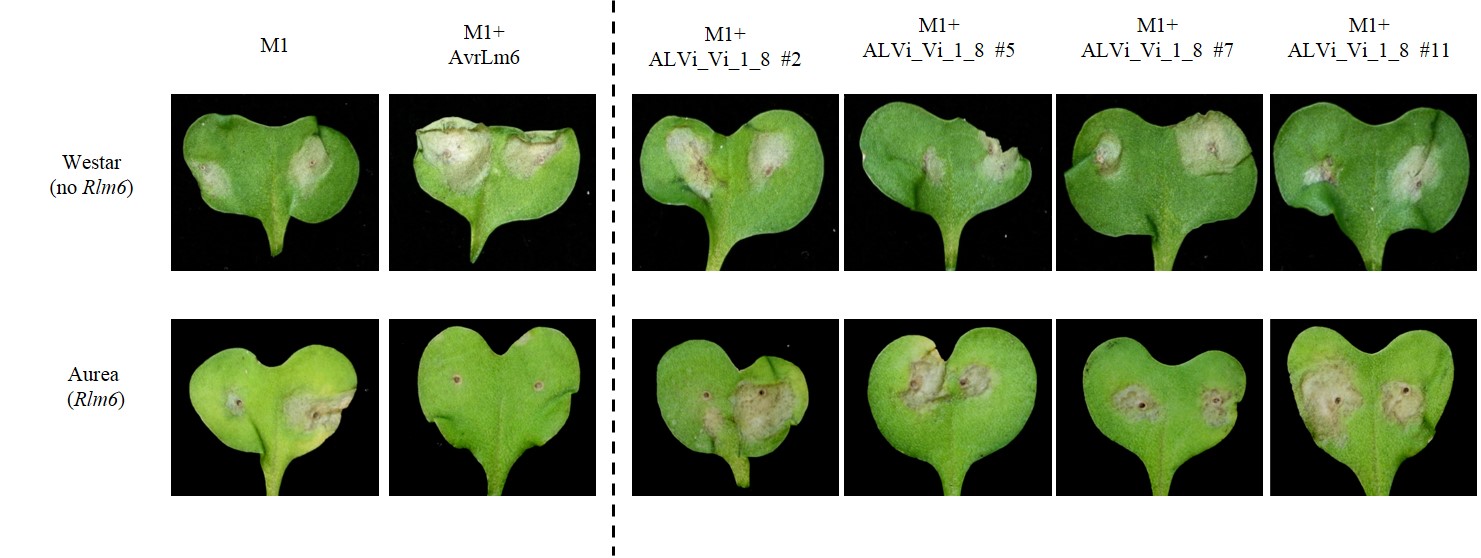


**Table S5**. *V. inaequalis* genes and the associated NCBI [eukaryotic clusters of orthologous groups](http://www.ncbi.nlm.nih.gov/COG/) (KOGs) IDs.

| **geneid** | **KOG ID** |
| --- | --- |
| atg4317 | KOG0002.4_1 |
| atg1783 | KOG0003.2_1 |
| atg12788 | KOG0018.4_1 |
| atg10670 | KOG0019.4_1 |
| atg2056 | KOG0025.3_1 |
| atg798 | KOG0047.2_1 |
| atg8605 | KOG0062.7_1 |
| atg10306 | KOG0073.4_1 |
| atg5714 | KOG0077.4_1 |
| atg13242 | KOG0084.10_1 |
| atg2392 | KOG0092.3_1 |
| atg11060 | KOG0094.4_1 |
| atg3029 | KOG0100.4_1 |
| atg4208 | KOG0102.2_1 |
| atg11656 | KOG0103.5_1 |
| atg4448 | KOG0122.4_1 |
| atg8347 | KOG0142.11_1 |
| atg7439 | KOG0173.1_1 |
| atg11837 | KOG0174.8_1 |
| atg10501 | KOG0175.9_1 |
| atg9193 | KOG0176.1_1 |
| atg5230 | KOG0177.1_1 |
| atg3019 | KOG0179.10_1 |
| atg2381 | KOG0180.2_1 |
| atg8413 | KOG0181.6_1 |
| atg658 | KOG0182.1_1 |
| atg1324 | KOG0183.3_1 |
| atg2377 | KOG0184.2_1 |
| atg3017 | KOG0185.11_1 |
| atg3315 | KOG0188.9_1 |
| atg13128 | KOG0190.6_1 |
| atg2853 | KOG0209.6_1 |
| atg1988 | KOG0211.9_1 |
| atg6182 | KOG0225.4_1 |
| atg10911 | KOG0233.3_1 |
| atg6903 | KOG0258.6_1 |
| atg6017 | KOG0261.1_1 |
| atg1567 | KOG0264.7_1 |
| atg1217 | KOG0271.5_1 |
| atg5665 | KOG0276.2_1 |
| atg3113 | KOG0279.7_1 |
| atg7058 | KOG0285.4_1 |
| atg12341 | KOG0289.12_1 |
| atg8966 | KOG0292.7_1 |
| atg8952 | KOG0302.7_1 |
| atg12708 | KOG0313.10_1 |
| atg5031 | KOG0318.4_1 |
| atg8478 | KOG0327.2_1 |
| atg9367 | KOG0328.4_1 |
| atg3569 | KOG0329.2_1 |
| atg4771 | KOG0330.9_1 |
| atg12991 | KOG0331.6_1 |
| atg5630 | KOG0344.3_1 |
| atg2455 | KOG0346.1_1 |
| atg8764 | KOG0357.1_1 |
| atg2262 | KOG0358.7_1 |
| atg13161 | KOG0359.7_1 |
| atg6721 | KOG0361.5_1 |
| atg6188 | KOG0362.2_1 |
| atg12718 | KOG0363.5_1 |
| atg3077 | KOG0364.3_1 |
| atg3720 | KOG0365.10_1 |
| atg12581 | KOG0366.12_1 |
| atg12569 | KOG0367.10_1 |
| atg12009 | KOG0371.1_1 |
| atg8608 | KOG0373.5_1 |
| atg13231 | KOG0376.7_1 |
| atg8937 | KOG0394.1_1 |
| atg8792 | KOG0397.5_1 |
| atg11033 | KOG0400.4_1 |
| atg10333 | KOG0402.6_1 |
| atg11526 | KOG0407.4_1 |
| atg8154 | KOG0418.4_1 |
| atg1700 | KOG0419.8_1 |
| atg930 | KOG0420.3_1 |
| atg11052 | KOG0424.7_1 |
| atg2607 | KOG0434.4_1 |
| atg10802 | KOG0441.2_1 |
| atg3689 | KOG0460.4_1 |
| atg8798 | KOG0466.6_1 |
| atg6951 | KOG0469.1_1 |
| atg10157 | KOG0477.4_1 |
| atg10847 | KOG0481.2_1 |
| atg6230 | KOG0495.5_1 |
| atg4593 | KOG0523.5_1 |
| atg1089 | KOG0524.4_1 |
| atg5117 | KOG0530.2_1 |
| atg1304 | KOG0534.3_1 |
| atg4569 | KOG0556.2_1 |
| atg6701 | KOG0559.6_1 |
| atg8143 | KOG0563.3_1 |
| atg6315 | KOG0567.4_1 |
| atg2737 | KOG0602.5_1 |
| atg12711 | KOG0622.11_1 |
| atg9267 | KOG0625.5_1 |
| atg12676 | KOG0631.4_1 |
| atg12573 | KOG0659.16_1 |
| atg1695 | KOG0675.11_1 |
| atg5609 | KOG0679.1_1 |
| atg9363 | KOG0683.8_1 |
| atg6902 | KOG0687.7_1 |
| atg9868 | KOG0688.4_1 |
| atg7420 | KOG0727.1_1 |
| atg1087 | KOG0728.2_1 |
| atg6035 | KOG0729.4_1 |
| atg8189 | KOG0741.3_1 |
| atg10971 | KOG0756.7_1 |
| atg979 | KOG0758.2_1 |
| atg12958 | KOG0767.11_1 |
| atg3302 | KOG0780.14_1 |
| atg8572 | KOG0784.3_1 |
| atg10123 | KOG0785.5_1 |
| atg2046 | KOG0787.4_1 |
| atg5749 | KOG0788.9_1 |
| atg8653 | KOG0815.8_1 |
| atg12775 | KOG0820.11_1 |
| atg1806 | KOG0829.13_1 |
| atg11138 | KOG0852.5_1 |
| atg2586 | KOG0853.5_1 |
| atg9892 | KOG0857.1_1 |
| atg4698 | KOG0861.4_1 |
| atg13007 | KOG0862.2_1 |
| atg12404 | KOG0871.9_1 |
| atg9138 | KOG0876.10_1 |
| atg9682 | KOG0878.13_1 |
| atg9451 | KOG0880.3_1 |
| atg3159 | KOG0888.1_1 |
| atg7916 | KOG0894.5_1 |
| atg4221 | KOG0898.4_1 |
| atg5898 | KOG0922.1_1 |
| atg7887 | KOG0927.1_1 |
| atg4437 | KOG0933.1_1 |
| atg12769 | KOG0934.6_1 |
| atg1717 | KOG0935.11_1 |
| atg5457 | KOG0937.9_1 |
| atg4359 | KOG0938.2_1 |
| atg11046 | KOG0948.2_1 |
| atg2010 | KOG0959.4_1 |
| atg7127 | KOG0960.4_1 |
| atg12620 | KOG0964.5_1 |
| atg8790 | KOG0985.10_1 |
| atg1703 | KOG0989.5_1 |
| atg13234 | KOG0991.11_1 |
| atg5700 | KOG0996.2_1 |
| atg4970 | KOG1036.6_1 |
| atg3854 | KOG1047.6_1 |
| atg6479 | KOG1058.1_1 |
| atg6603 | KOG1062.8_1 |
| atg2314 | KOG1068.3_1 |
| atg684 | KOG1077.1_1 |
| atg11685 | KOG1078.9_1 |
| atg3012 | KOG1088.10_1 |
| atg10805 | KOG1098.8_1 |
| atg3653 | KOG1099.2_1 |
| atg4274 | KOG1112.6_1 |
| atg10447 | KOG1123.4_1 |
| atg4818 | KOG1131.3_1 |
| atg3149 | KOG1137.5_1 |
| atg2785 | KOG1145.4_1 |
| atg4255 | KOG1149.11_1 |
| atg2331 | KOG1158.3_1 |
| atg10329 | KOG1159.5_1 |
| atg3852 | KOG1180.1_1 |
| atg1232 | KOG1211.8_1 |
| atg11528 | KOG1235.5_1 |
| atg5322 | KOG1241.3_1 |
| atg4360 | KOG1255.2_1 |
| atg3011 | KOG1268.10_1 |
| atg8053 | KOG1272.3_1 |
| atg11339 | KOG1291.6_1 |
| atg13062 | KOG1299.7_1 |
| atg2845 | KOG1301.3_1 |
| atg7631 | KOG1322.7_1 |
| atg5951 | KOG1335.8_1 |
| atg743 | KOG1342.2_1 |
| atg7823 | KOG1349.2_1 |
| atg12896 | KOG1350.8_1 |
| atg5047 | KOG1351.3_1 |
| atg436 | KOG1353.3_1 |
| atg11289 | KOG1355.9_1 |
| atg9508 | KOG1358.7_1 |
| atg9532 | KOG1367.5_1 |
| atg4755 | KOG1370.11_1 |
| atg3192 | KOG1373.5_1 |
| atg12973 | KOG1374.9_1 |
| atg10836 | KOG1390.5_1 |
| atg9226 | KOG1393.3_1 |
| atg5588 | KOG1394.5_1 |
| atg11068 | KOG1415.6_1 |
| atg3336 | KOG1430.3_1 |
| atg11502 | KOG1433.3_1 |
| atg1589 | KOG1439.2_1 |
| atg9073 | KOG1448.2_1 |
| atg5790 | KOG1458.1_1 |
| atg2873 | KOG1463.11_1 |
| atg2513 | KOG1466.5_1 |
| atg6922 | KOG1468.2_1 |
| atg11779 | KOG1487.6_1 |
| atg292 | KOG1491.1_1 |
| atg1794 | KOG1494.6_1 |
| atg11141 | KOG1498.2_1 |
| atg6859 | KOG1506.10_1 |
| atg7470 | KOG1523.4_1 |
| atg3100 | KOG1526.8_1 |
| atg3362 | KOG1531.3_1 |
| atg2192 | KOG1532.10_1 |
| atg10658 | KOG1533.11_1 |
| atg6858 | KOG1534.10_1 |
| atg5834 | KOG1535.10_1 |
| atg2742 | KOG1540.9_1 |
| atg5890 | KOG1541.9_1 |
| atg7517 | KOG1549.1_1 |
| atg742 | KOG1555.2_1 |
| atg8312 | KOG1556.8_1 |
| atg12946 | KOG1562.12_1 |
| atg10853 | KOG1566.4_1 |
| atg7356 | KOG1567.4_1 |
| atg5062 | KOG1568.1_1 |
| atg12280 | KOG1596.6_1 |
| atg12709 | KOG1597.6_1 |
| atg191 | KOG1626.5_1 |
| atg179 | KOG1636.1_1 |
| atg3272 | KOG1641.13_1 |
| atg8948 | KOG1643.7_1 |
| atg9977 | KOG1644.1_1 |
| atg9080 | KOG1646.3_1 |
| atg7783 | KOG1647.5_1 |
| atg13211 | KOG1654.15_1 |
| atg8051 | KOG1662.3_1 |
| atg2576 | KOG1664.3_1 |
| atg3932 | KOG1668.5_1 |
| atg8291 | KOG1678.10_1 |
| atg11232 | KOG1688.9_1 |
| atg1697 | KOG1691.9_1 |
| atg12084 | KOG1692.7_1 |
| atg9422 | KOG1712.4_1 |
| atg10226 | KOG1722.6_1 |
| atg5330 | KOG1723.1_1 |
| atg1523 | KOG1727.5_1 |
| atg3167 | KOG1728.2_1 |
| atg9107 | KOG1733.3_1 |
| atg1930 | KOG1742.10_1 |
| atg1150 | KOG1746.3_1 |
| atg5686 | KOG1750.4_1 |
| atg963 | KOG1753.1_1 |
| atg12516 | KOG1754.14_1 |
| atg3926 | KOG1755.9_1 |
| atg2811 | KOG1758.4_1 |
| atg2178 | KOG1760.6_1 |
| atg3695 | KOG1762.5_1 |
| atg5919 | KOG1769.7_1 |
| atg11099 | KOG1770.12_1 |
| atg8002 | KOG1772.10_1 |
| atg339 | KOG1774.4_1 |
| atg8349 | KOG1775.12_1 |
| atg2333 | KOG1779.1_1 |
| atg6034 | KOG1780.4_1 |
| atg6486 | KOG1781.2_1 |
| atg6789 | KOG1782.11_1 |
| atg3962 | KOG1784.8_1 |
| atg10115 | KOG1795.5_1 |
| atg3280 | KOG1800.14_1 |
| atg13067 | KOG1816.7_1 |
| atg9010 | KOG1872.10_1 |
| atg4084 | KOG1885.3_1 |
| atg2393 | KOG1889.6_1 |
| atg10010 | KOG1915.4_1 |
| atg10637 | KOG1936.7_1 |
| atg12466 | KOG1942.11_1 |
| atg10641 | KOG1979.9_1 |
| atg9089 | KOG1980.5_1 |
| atg4572 | KOG1986.3_1 |
| atg13314 | KOG1992.13_1 |
| atg669 | KOG2004.3_1 |
| atg7883 | KOG2014.1_1 |
| atg9816 | KOG2017.5_1 |
| atg11361 | KOG2035.5_1 |
| atg347 | KOG2036.1_1 |
| atg5424 | KOG2044.5_1 |
| atg7784 | KOG2047.4_1 |
| atg12334 | KOG2067.12_1 |
| atg8121 | KOG2104.13_1 |
| atg9854 | KOG2189.7_1 |
| atg3859 | KOG2270.2_1 |
| atg8290 | KOG2276.8_1 |
| atg6714 | KOG2292.5_1 |
| atg3959 | KOG2303.11_1 |
| atg7197 | KOG2309.8_1 |
| atg5440 | KOG2321.11_1 |
| atg5378 | KOG2387.3_1 |
| atg691 | KOG2415.6_1 |
| atg11464 | KOG2446.17_1 |
| atg12825 | KOG2451.8_1 |
| atg7423 | KOG2467.1_1 |
| atg1987 | KOG2472.5_1 |
| atg5073 | KOG2481.1_1 |
| atg5256 | KOG2509.1_1 |
| atg1721 | KOG2519.5_1 |
| atg745 | KOG2529.2_1 |
| atg12299 | KOG2531.5_1 |
| atg5390 | KOG2535.7_1 |
| atg3923 | KOG2537.4_1 |
| atg5577 | KOG2555.3_1 |
| atg2215 | KOG2572.6_1 |
| atg10009 | KOG2574.6_1 |
| atg10298 | KOG2575.1_1 |
| atg10896 | KOG2606.5_1 |
| atg7466 | KOG2613.2_1 |
| atg11754 | KOG2617.6_1 |
| atg4625 | KOG2623.2_1 |
| atg9368 | KOG2636.6_1 |
| atg8619 | KOG2638.6_1 |
| atg4544 | KOG2653.1_1 |
| atg8795 | KOG2670.9_1 |
| atg9180 | KOG2680.1_1 |
| atg3354 | KOG2700.2_1 |
| atg4658 | KOG2703.5_1 |
| atg10519 | KOG2707.11_1 |
| atg12177 | KOG2711.14_1 |
| atg2240 | KOG2719.6_1 |
| atg9724 | KOG2726.12_1 |
| atg7679 | KOG2728.3_1 |
| atg9462 | KOG2732.13_1 |
| atg2642 | KOG2738.7_1 |
| atg9980 | KOG2749.2_1 |
| atg4853 | KOG2754.8_1 |
| atg882 | KOG2757.2_1 |
| atg10321 | KOG2767.4_1 |
| atg10482 | KOG2770.12_1 |
| atg6111 | KOG2772.4_1 |
| atg11719 | KOG2775.4_1 |
| atg885 | KOG2781.3_1 |
| atg6367 | KOG2783.3_1 |
| atg1193 | KOG2784.2_1 |
| atg3663 | KOG2785.3_1 |
| atg6358 | KOG2792.4_1 |
| atg8060 | KOG2803.7_1 |
| atg7253 | KOG2807.14_1 |
| atg3235 | KOG2825.4_1 |
| atg4510 | KOG2833.1_1 |
| atg1120 | KOG2851.4_1 |
| atg3974 | KOG2854.10_1 |
| atg3474 | KOG2855.3_1 |
| atg3750 | KOG2874.9_1 |
| atg56 | KOG2877.1_1 |
| atg8410 | KOG2906.13_1 |
| atg4737 | KOG2908.8_1 |
| atg10483 | KOG2909.11_1 |
| atg8235 | KOG2916.8_1 |
| atg3191 | KOG2930.3_1 |
| atg11547 | KOG2948.13_1 |
| atg11287 | KOG2952.12_1 |
| atg7626 | KOG2957.3_1 |
| atg6696 | KOG2967.6_1 |
| atg11177 | KOG2971.8_1 |
| atg11803 | KOG2981.9_1 |
| atg9891 | KOG2988.1_1 |
| atg8482 | KOG3013.4_1 |
| atg8585 | KOG3022.13_1 |
| atg2134 | KOG3031.2_1 |
| atg4374 | KOG3048.4_1 |
| atg12064 | KOG3049.9_1 |
| atg2370 | KOG3052.5_1 |
| atg17 | KOG3064.1_1 |
| atg3347 | KOG3079.3_1 |
| atg5041 | KOG3090.2_1 |
| atg8323 | KOG3106.10_1 |
| atg3095 | KOG3147.10_1 |
| atg11219 | KOG3157.9_1 |
| atg9621 | KOG3163.2_1 |
| atg8643 | KOG3164.11_1 |
| atg2748 | KOG3167.1_1 |
| atg8299 | KOG3174.12_1 |
| atg9117 | KOG3180.3_1 |
| atg11469 | KOG3185.16_1 |
| atg5145 | KOG3188.2_1 |
| atg5118 | KOG3189.3_1 |
| atg11628 | KOG3204.15_1 |
| atg3610 | KOG3205.9_1 |
| atg7073 | KOG3218.4_1 |
| atg4313 | KOG3222.5_1 |
| atg3263 | KOG3229.8_1 |
| atg12402 | KOG3232.5_1 |
| atg2542 | KOG3237.1_1 |
| atg1895 | KOG3239.4_1 |
| atg9803 | KOG3271.10_1 |
| atg12180 | KOG3273.10_1 |
| atg4946 | KOG3275.3_1 |
| atg4976 | KOG3283.10_1 |
| atg7136 | KOG3284.5_1 |
| atg6054 | KOG3285.5_1 |
| atg6668 | KOG3291.2_1 |
| atg6433 | KOG3295.7_1 |
| atg4232 | KOG3297.9_1 |
| atg11007 | KOG3301.12_1 |
| atg2181 | KOG3311.8_1 |
| atg7448 | KOG3313.7_1 |
| atg4580 | KOG3318.10_1 |
| atg1849 | KOG3320.6_1 |
| atg3081 | KOG3330.11_1 |
| atg4017 | KOG3343.12_1 |
| atg3114 | KOG3349.19_1 |
| atg8210 | KOG3361.10_1 |
| atg7021 | KOG3387.4_1 |
| atg1110 | KOG3400.3_1 |
| atg2486 | KOG3404.3_1 |
| atg2725 | KOG3405.6_1 |
| atg5336 | KOG3406.6_1 |
| atg3231 | KOG3411.13_1 |
| atg2874 | KOG3418.5_1 |
| atg5084 | KOG3428.4_1 |
| atg10786 | KOG3430.6_1 |
| atg487 | KOG3432.1_1 |
| atg3931 | KOG3436.8_1 |
| atg11214 | KOG3442.8_1 |
| atg1897 | KOG3448.11_1 |
| atg4220 | KOG3449.4_1 |
| atg4954 | KOG3453.6_1 |
| atg9116 | KOG3457.2_1 |
| atg2400 | KOG3459.5_1 |
| atg10912 | KOG3463.4_1 |
| atg10869 | KOG3464.7_1 |
| atg6715 | KOG3475.10_1 |
| atg9678 | KOG3479.10_1 |
| atg4723 | KOG3480.7_1 |
| atg7066 | KOG3482.3_1 |
| atg9878 | KOG3489.14_1 |
| atg8445 | KOG3493.18_1 |
| atg7922 | KOG3497.11_1 |
| atg5805 | KOG3498.3_1 |
| atg3013 | KOG3499.12_1 |
| atg10443 | KOG3502.7_1 |
| atg5463 | KOG3503.18_1 |
| atg6162 | KOG3506.8_1 |
| atg271 | KOG3855.4_1 |
| atg2963 | KOG3954.6_1 |
| atg230 | KOG3974.3_1 |
| atg4007 | KOG4392.13_1 |
| atg5030 | KOG4655.2_1 |
